# Insulin Infusion on Postoperative Complications of Coronary Artery Bypass Graft in Patients With Diabetes Mellitus

**DOI:** 10.5812/cardiovascmed.17861

**Published:** 2014-04-01

**Authors:** Gholamreza Masoumi, Rasoul Frasatkhish, Hamid Bigdelian, Mohsen Ziyaefard, Ali Sadeghpour-Tabae, Mojtaba Mansouri, Alireza Jalali

**Affiliations:** 1 Department of Anesthesiology, Isfahan University of Medical Sciences, Isfahan, IR Iran; 2 Rajaie Cardiovascular Medical and Research Center, Iran University of Medical Sciences, Tehran, IR Iran; 3 Department of Cardiac Surgery, Isfahan University of Medical Sciences, Isfahan, IR Iran; 4 Department of Anesthesiology, Baqiyatallah University of Medical Sciences, Tehran, IR Iran

**Keywords:** Insulin, Coronary Artery Bypass Graft, Glucose, Surgery

## Abstract

**Background:**

Cardiovascular events are common in patients with diabetes mellitus (DM), which make coronary artery bypass graft (CABG) a highly demanded surgery in this population. Tight control of blood glucose in patients with DM is beneficial in reducing postoperative complications; however, the adequate range has not been determined yet.

**Objectives:**

This study aimed to investigate the effect of semi-tight (moderate) control of DM on complications and serum glucose levels during and after CABG.

**Patients and Methods:**

In this prospective clinical trial, 18 and 31 patients with and without DM, respectively, who were referred to Shahid Chamran Hospital, Isfahan, Iran, for elective CABG surgery, were enrolled. For DM group, patients with controlled DM (i.e. glycosylated hemoglobin levels [HgA1C] ≤ 7%) were recruited. Blood glucose level (blood sugar, BS) was measured after anesthesia, during pumping, warming, off pumping, six and 12 hours after Intensive Care Unit (ICU) admission, and at discharging from the hospital. The hemodynamic state of the patients, bleeding, need of blood transfusion, infection, and duration of hospitalization were also monitored and recorded.

**Results:**

None of the BS measurements (FBS, after anesthesia, on-pump, warming, off pump, six and 12 hours after ICU admission, and at discharge) were significantly different between study groups (P > 0.05). Frequency of surgery site bleeding and blood transfusion need were not significantly different between these groups (P > 0.05).

**Conclusions:**

Semi-tight control of DM with insulin infusion during operation did not led to any difference in the type and rate of CABG complications between patients with well-controlled and those without DM; however, BS levels in patients with well-controlled DM could be more easily controlled.

## 1. Background

Diabetes mellitus (DM) is the most prevalent metabolic disorder and one of the most important causes of mortality and morbidity worldwide. Almost 25% of patients do not have insight to their disease unless the disorder manifests with one of its complications (1). A study has shown that 50% of all patients are affected by at least one of the DM complications (2). However, nowadays with great advances in diagnostic and management technologies, more patients are considered as good candidates for surgery. Vitrectomy, cataract, wound debridement, kidney transplant, and vessels repair microsurgeries are the most prevalence surgeries in patients with DM (3). Cardiovascular events are the most important cause of death in patients with DM with three to seven folds higher risk in comparison to general population. Cardiac surgeries are also frequent to increase survival and restore the quality of life. Therefore, request for surgeries are increasing among patients with DM, which are necessaryin most cases, (4, 5). As a highly demanded surgery, coronary artery bypass graft (CABG) can put both healthcare receivers and providers in jeopardy of complications such as infections and high glucose levels before, during, and after surgery. Different studies have focused on surgery complication and most of them demonstrated more prevalent surgery site infections (6, 7). According to researchers, tight control of glucose in patients with DM is beneficial in reducing postoperation complications; however, the adequate range has not yet been defined (8, 9). Van den Berghe et al. have demonstrated that insulin infusion during surgery can maintain the glucose level between 80 to 110 mg/dL (10). Other studies have had similar results. Furthermore, there are studies showing that hyperglycemia can increase the in-hospital cardiovascular-related mortality rate in patients with myocardial infarction (10-13).

## 2. Objectives

Previous studies have shown that DM control can increase the success rate of cardiac surgery; however, the results remain unclear due to limitations such as complications evaluation. Therefore, this study aimed to investigate the effect of semi-tight control of blood glucose on complications and serum glucose levels during and after CABG surgery in patients with DM.

## 3. Patients and Methods

In this prospective nonrandomized clinical trial, 49 patients with American Stroke Association (ASA) class I, II, and III referred to Shahid Chamran Hospital, Isfahan, Iran, for elective CABG surgery, recruited. Eighteen patients with controlled DM (i.e. glycosylated hemoglobin levels [HgA1C] ≤ 7%) and 31 patients without DM (control group) were enrolled. All patients with ASA class IV or higher, a history of heart valves repair, kidney or liver diseases, and those undergoing emergent CABG were excluded. All patients underwent echocardiography and HgA1C was measured two days before surgery. Fasting blood sugar (FBS) was measured in the morning of the surgery day. They were under cardiac monitoring, pulse-oximetry, periodic electrocardiography, capnography, and invasive blood pressure monitoring during operation. Balanced anesthesia was induced in all patients by sufentanil, midazolam, and propofol. All patients in DM group underwent semi-tight (moderate) glycemic control (FBS of 90 - 150 mg/dL) with continious infusion of 50 IU regular insulin in 500 mL of dextrose 10% in water during surgery in a range of 1 IU/h to 5 IU/h insulin. Insulin infusion was started after anesthesia induction and continued until discharging from Intensive Care Unit (ICU). Patients in control group received routine cares. We measured BS in all patients after anesthesia, during pumping, warming, off-pumping, six as well as 12 hours after ICU admission, and at the time of discharging from the hospital; moreover, the hemodynamic state of the patients, bleeding, need to blood transfusion, infection, and duration of hospitalization were evaluated. 

All patients signed an informed consent before enrollment. Participants could exit from the study whenever they wanted. The protocol of the study was approved by Isfahan University of Medical Sciences Ethics Committee (research project number 36189).

### 3.1. Statistical Analysis

The data were analyzed using SPSS 15 for Windows (SPSS Inc., Chicago, IL, USA). Data were expressed as mean ± standard deviation (SD) for interval and count (%) for categorical variables. Student t-test was used for comparison of continuous variables with normal distribution and Mann-Whitney U-test was used for nonparametric comparison between two groups. Chi-square test was used to compare proportions of categorical variables between two groups. P value less than 0.05 was considered as the statistically significant.

## 4. Results

### 4.1. Baseline Characteristics

Forty-nine patients including 18 patients with controlled DM and 31 patients without DM were recruited. None of the assessed patients were excluded before CABG. The mean age of DM and control group was 56.1 ± 11.3 and 58.3 ± 9.4 years, respectively, and there was no significant difference between the two groups with regard to age (P = 0.661). The male to female ratio was 44.3/55.7% in the DM and 67.3/32.7% in the control groups (P = 0.118). Although mean ICU stay in the DM group was slightly longer than in the control group, the difference was not statistically significant (P = 0.811), (Table 1). Moreover, mean duration of surgery and pump application were not significantly different between the study groups (P value of 0.53 and 0.09, respectively; Table 1). Mean preoperative ejection fraction percentage of the control and DM groups were 43.06 ± 11.0 and 44.70 ± 12.70, respectively, which had no significant difference (P = 0.373). Measurements of BS before and after anesthesia, on-pump, and six hours after ICU admission showed significant difference between study groups (P < 0.05). In other study points (warming, off pump, 24 hours after ICU admission, and at discharge from ICU), BS was not significantly different between groups (P > 0.05, Table 1). Prevalence of bleeding from the surgery site and rate of the need for blood transfusion did not show any significant difference betweengroups at any study point (P > 0.05), except for 24 hours after ICU admission that transfusion need was significantly more among patients with DM (P = 0.01). There was no significant difference between the patients with controlled DM and patients without DM with regard to other complications such as respiratory, gastrointestinal, and heart disorders, as well as sepsis, need for inotropic drugs or assisted device, reoperation rate, and CCU/ICU readmission rate.

**Table 1.** Demographic and Measurement Data in Both Groups ^a^

Characteristics	Non-diabetic Group (n=31)	Controlled Diabetic Group (n=18)	P value
**Gender, %**			0.118
Male	67.3	44.3	
Female	32.7	55.7	
**Age, y**	58.3 ± 9.4	56.1 ± 11.3	0.661
**Ejection fraction, %**	43.06 ± 11.0	44.70 ± 12.70	0.373
**Duration of surgery, min**	271.93 ± 50.55	283.33 ± 66.95	0.53
**Duration of pump application, min**	74.03 ± 26.93	91.61 ± 37.44	0.09
**BS before inducing anesthesia, mg/dL** ^**b**^	101.88 ± 20.39	144.46 ± 44.02	0.002
**BS after inducing anesthesia, mg/dL**	97.58 ± 29.27	136.78 ± 48.49	0.014
**BS during pump, mg/dL**	136.38 ± 25.05	155.06 ± 35.36	0.040
**BS at warming, mg/dL**	163.59 ± 38.05	176.00 ± 43.52	0.317
**BS at off pump, mg/dL**	167.69 ± 37.52	189.61 ± 53.15	0.138
**BS after 6 hours in ICU, mg/dL**	186.03 ± 69.02	235.00 ± 85.02	0.048
**BS after 24 hours in ICU, mg/dL**	163.78 ± 48.35	187.78 ± 68.40	0.208
**BS at discharge, mg/dL**	135.33 ± 39.87	170.79 ± 44.41	0.260
**ICU admission duration, d**	2.60 ± 0.67 3	44 ± 2.25	0.811
**Infection, count (%)**	5 (9.6)	3 (5.8)	0.313
**Bleeding at surgery site, count (%)**	6 (19.4)	2 (11.1)	0.452
**Blood transfusion need, count (%)**	3 (5.8)	6 (11.5)	0.132

^a^Data are presented as Mean ± SD.

^b^Abbreviations: BS, blood sugar (glucose); ICU, intensive care unit.

## 5. Discussion

We assessed the effect of insulin infusion on serum glucose during and after CABG in patients without DM and with controlled DM, which remained stable in a range of 150 to 180 mg/dL. Our results showed that significant difference was found in BS between these groups; however, BS in control group was more easily controlled during the surgery. In comparison with other studies results, none of the patients underwent semi-tight control. Instead, the patients had tight-controlled infusion or non-tight control during the surgery (12-15). The mean admission time in the ICU was nearly three days in both groups without significant difference. In two different studies by Paun et al. and Bucerius et al., which studied ICU admission duration in patients with DM after CABG, no significant difference was found between DM and control groups. However, this duration was much longer in their studies. Although in Cardiac-ICUs, the BS is controlled between 200 to 220 mg/dL (16, 17), we have intervened in patients with BS > 180 mg/dL to see whether the rate of complications would be less in blood suger less than 180 mg/dL. Our study also demonstrated no significant difference with regard to complications such as bleeding, need of blood transfusion, and infection between study groups. Similarly, Tsuruta et al. (18) found no difference in infection rate and Wallin et al. (19) demonstrated no difference in bleeding prevalence between patients with and without DM. Our study results showed that the complications of the CABG did not differ in type and rate in patients with controlled DM infused by insulin during surgery with controls. We recommend more studies with greater sample size and longer follow-up duration to confirm our results as they were our limitations in this study.
